# Supplementary material for: Preparation and Properties of Starch–Cellulose Composite Aerogel
Source: Polymers (Basel). 2023 Nov 1;15(21):4294. doi: 10.3390/polym15214294 (PMC10648849; doi:10.3390/polym15214294)
Supplement: Supplementary file 1 [file polymers-15-04294-s001.zip › polymers-2603165-supplementary.pdf]

Supplementary File

Preparation and properties of starch-cellulose composite aerogel

Jihong Huang 1, Jingyang Gao 2 , Liang Qi 2 , Qunyu Gao 2* and Ling Fan 1*

1 *College Food and Pharmacy College, Xuchang University, Xuchang, Henan 461000*

2 *School of Food Science and Engineering, South China University of Technology, Guangzhou, Guangdong 510640*

[*]*Corresponding author:*

Qunyu Gao, Tel/Fax: +86-20-87113845, E-mail: qygao@scut.edu.cn.

Jihong Huang, Tel/Fax: +86-373-67758022, E-mail: huangjih1216@126.com

Synthesis mechanism of cross-linking starch by MBA

(1) The initiator decomposes to generate free radicals：

$$S_2O_8^{2-} + HSO_3^{-} \longrightarrow SO_4^{2-} + SO_4^{-}\cdot + HSO_3\cdot \qquad (S1)$$

$$SO_4^{-}\cdot + H_2O \longrightarrow HSO_4^{-} + OH\cdot \qquad (S2)$$

The radicals $SO_4^{-}\cdot$, $HSO_3\cdot$ and $OH\cdot$ are all denoted as $R\cdot$.

(2) Copolymerization of MBA and Starch:

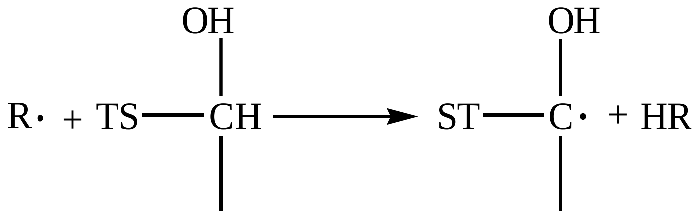

(S3)

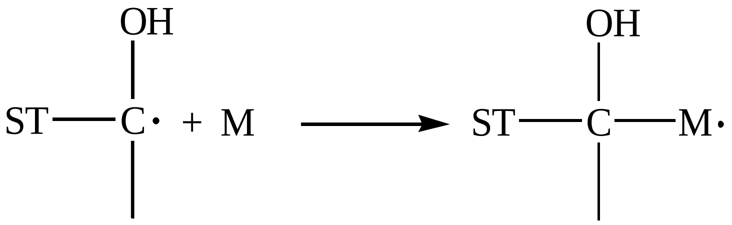

(S4)

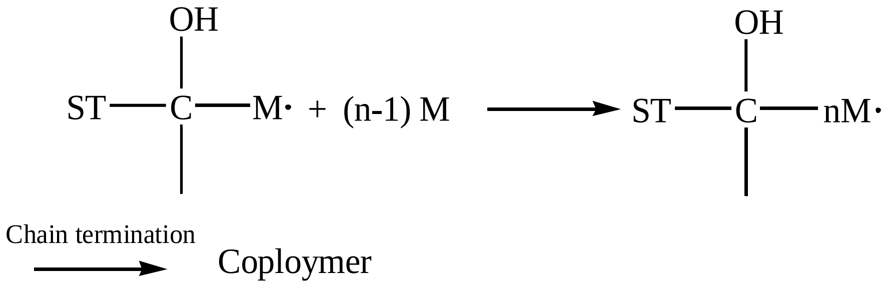

(S5)

where M represents MBA monomer, 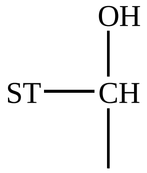

is the starch molecule.

Note: According to our previous study, the C_2, C_3 and C_6 in the anhydroglucose were all involved in the copolymerization. Meanwhile, since there are two unstable double bonds in the MBA molecule, it has higher copolymerization reactivity. The possible structure of the copolymers formed by starch and MBA are shown as follows:

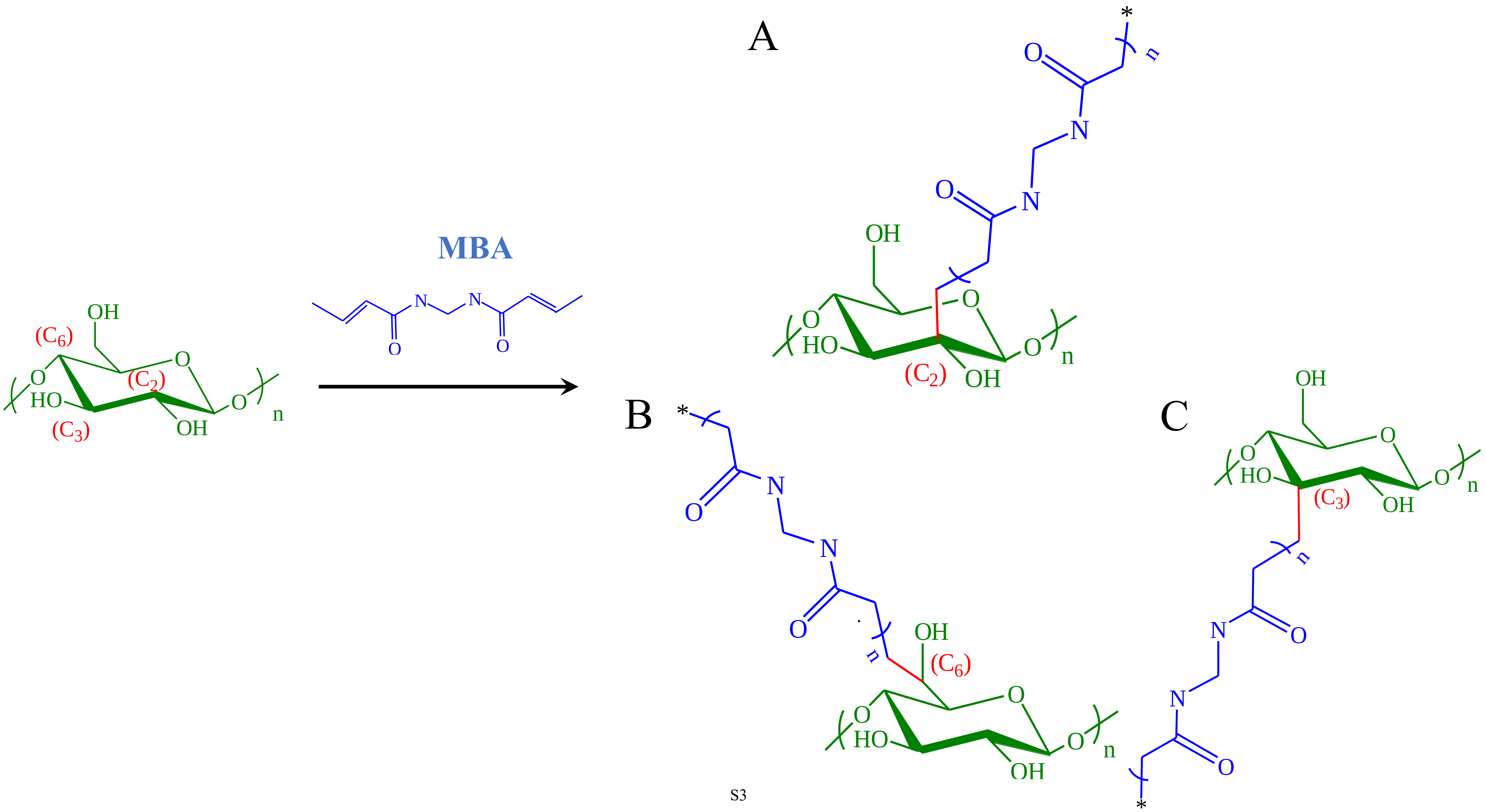

Figure S1. Schematic diagram of the copolymers formed by starch and MBA at (A) C2, (B) C3 and (C) C6 in the anhydroglucose

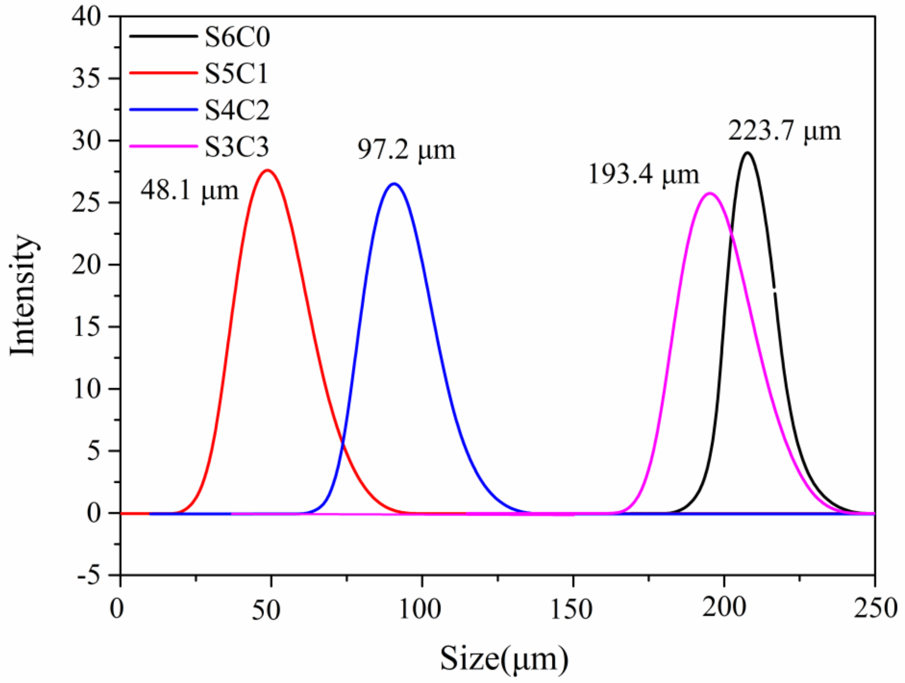

Figure S2 size distribution of S6C0, S5C1 aerogel, S4C2 aerogel and S3C3 aerogel

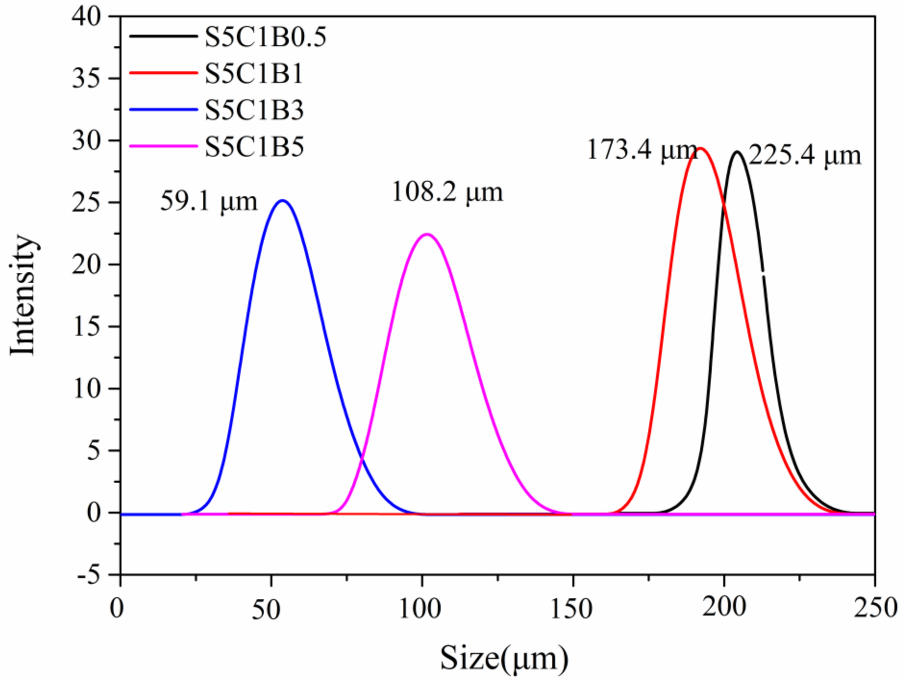

Figure S3 size distribution of S5C1B0.5 aerogel, S5C1B1 aerogel, S5C1B3 aerogel, and S5C1B5 aerogel
